# Ecological niche modeling predicting the potential distribution of *Leishmania* vectors in the Mediterranean basin: impact of climate change

**DOI:** 10.1186/s13071-018-3019-x

**Published:** 2018-08-09

**Authors:** Bilel Chalghaf, Jomâa Chemkhi, Benjamin Mayala, Myriam Harrabi, Goze Bertin Benie, Edwin Michael, Afif Ben Salah

**Affiliations:** 1 Pasteur Institute of Tunis, Tunis, Tunisia; 2 Le Centre d’Enseignement et de Recherche en Foresterie de Ste-Foy, Québec, Canada; 3 The Centre for Research and Applications in Remote Sensing, Department of Applied Geomatics, Sherbrooke University, Sherbrooke, Quebec Canada; 4 University of Notre Dame, Indiana, USA; 5 Department of Family and Community Medicine, Arabian Gulf University, Manama, Bahrain

**Keywords:** Leishmaniasis, Vectors, Geographical distribution, Modeling, Climate change

## Abstract

**Background:**

Due to climate change, the geographical distribution of sand flies during the last decades has shifted northward from latitudes below 45°N in southern Europe to latitudes just above 50^○^N. Recent studies show that some phlebotomine sand flies were recorded in several parts of Germany and Belgium. In central Europe, some autochthone leishmaniasis cases are being recorded in regions traditionally regarded as leishmaniasis-free. An important challenge is to predict the geographical distribution of leishmaniasis vectors under new climatic conditions. In this study, we attempted to predict the current distribution of six leishmaniasis vectors in the Mediterranean basin and forecast species’ geographical shift under future climate scenarios using an ensemble ecological niche modeling approach. Species records were obtained from scientific surveys published in the research literature between 2006 and 2016. A series of climate metrics describing temperature and precipitation in the study area under two climatic scenarios were obtained from WorldClim database. A consensus model was derived from six varieties of modeling approaches (regression, machine learning and classification techniques) in order to ensure valid prediction of distribution of vectors under different climate scenarios.

**Results:**

Model performance was generally high for the included species with a specificity (true negative rate) ranging from 81.03 to 96.52% (mean = 86.94%) and a sensitivity (true positive rate) ranging from 87.93 to 100% (mean = 96.98%). Our work evidenced the hypothesis of the widespread of *Leishmania* vectors under climate change scenarios. All of the studied species are prospected to gain new areas that are actually not suitable for vectors’ survival. Phlebotomine sand flies are prospected to invade extra-Mediterranean regions, especially western and central Europe.

**Conclusions:**

Our study confirmed the importance of environmental and climate factors on the distribution of leishmaniasis vectors and demonstrated the performance of ecological niche modeling in the prediction of the geographical spread of vector-borne diseases. Ecological niche modeling should be considered in the future as a valuable tool in addition to experimental laboratory studies for a better understanding of the biology of vector species.

## Background

Global warming and climate change due to industrialization, intensive farming and anthropogenic activities leading to higher greenhouse gases emission, has become a fact. The direct consequences of increasing the greenhouse effect are changes in temperature, precipitation patterns and other climate variables [1].

The Mediterranean basin is considered one of the most vulnerable regions of the world to climate change [2]. Indeed, the Mediterranean region has shown large climate variation in the past [3] and has been identified as one of the most sensitive areas according to future climate change projections [4, 5]. According to the Intergovernmental Panel on Climate Change [1] temperature will increase in the Mediterranean area while annual precipitation will decrease during the 21st century. Indeed, annual rainfall will decrease in Mediterranean Africa and the northern Sahara, with a greater decrease as the Mediterranean coast is approached. Precipitation will increase in most of northern Europe and decrease in most southern countries bordering the Mediterranean Sea. The same projection for the 21st century states that the annual mean warming will vary from 2.2 to 5.1 °C in southern Europe and the Mediterranean area, leading to warmer summers and more temperate winters.

In addition to its direct effect on human health and welfare, consisting of excessive heat exposure and extreme weather events (storms, floods and droughts), climate change may also affect the spread of vector-borne diseases and other infectious diseases [6–8].

The environmental factors are the main causes of the emergence and re-emergence of infectious diseases. Without a doubt, environmental change can create favorable conditions for the proliferation of reservoirs, increase the density of vectors, and consolidate the reservoir-vector-host interaction [9]. Changes in climatic conditions (temperature, rainfall and humidity) that are prospected to occur under different climate change scenarios will affect the geographical distribution of vector-borne disease [10].

Leishmaniasis is a vector-borne infection caused by parasitic protozoans of the genus *Leishmania* that are transmitted to humans through the bite of an infected female phlebotomine sand fly. Among over 800 species of sand fly, 98 are proven or suspected vectors of human leishmaniases; these include 42 *Phlebotomus* species in the Old World and 56 *Lutzomyia* species in the New World [11]. In countries bordering the Mediterranean Sea, including those in southern Europe, North Africa and the Middle East, more than 20 species of *Phlebotomus* are widely distributed [12].

The optimal temperature for the development of sand flies and *Leishmania* parasites is approximately 25 °C [13, 14]. The prospected change in ambient temperature would increase the vector biting rate and number of human exposures, and reduce the incubation time of the infective agent within its vector [15, 16]. Furthermore, a higher temperature will reduce the vector winter mortality and new areas will become environmentally suitable for its survival and reproduction. There is evidence that the geographical range of phlebotomine sand flies incriminated in leishmaniasis transmission has changed in response to climate change in the Mediterranean region and Europe.

The geographical distribution of sand flies during the last decades has shifted northward from latitudes below 45°N in southern Europe [17] to latitudes just above 50°N [18]. Recent studies show that some phlebotomine sand flies were recorded in several parts of Germany and Belgium [18, 19]. In central Europe, some autochthone leishmaniasis cases are being recorded in a region traditionally regarded as leishmaniasis-free [20, 21].

Recently, species distribution modeling has acquired importance, not only for the ability to predict species’ geographical distribution from explanatory variables, but also for the faculty to forecast species’ occurrence under new areas and climatic conditions [22]. However, selecting the appropriate model is challenging not only for the various techniques available but also for the different approaches adopted by each model which yield to different results [23]. One alternative to address inter-model variations is to combine several models. The use of an ensemble approach can result in significant improvements on the robustness of predicting species distribution and forecasting their geographical shift under climate change projections [23].

The main motivation of this study was to (i) predict the current distribution of leishmaniasis vectors in the Mediterranean basin; (ii) forecast species’ geographical shifts under future climate scenarios; and (iii) evaluate climatic variables leading to the geographical spread or extinction of these vectors using an ensemble modeling approach.

## Methods

### Species data

Species records were obtained from scientific surveys published in research literature between 2006 and 2016 [24–37]. We performed a literature search in the SCOPUS database using search topic = *Phlebotomus* OR Sand flies OR Phlebotominae AND Geographical coordinate. Literature indicating only the name of the city or village where the species was recorded were discarded and only species’ occurrences with explicit geographical coordinates were retained. Since the values of environmental predictors at these locations were extracted to fit the model prediction of the species presence across the study area, integrating accurate geographical occurrence points is crucial for better model fitting.

We found a total of 384 data-points distributed in 16 Mediterranean countries (Albania, Algeria, Cyprus, Egypt, France, Greece, Israel, Italy, Lebanon, Libya, Montenegro, Morocco, Portugal, Spain, Tunisia and Turkey) and six phlebotomine sand fly species (27 for *Phlebotomus alexandri*; 66 for *Phlebotomus ariasi*; 25 for *Phlebotomus kazeruni*; 179 for *Phlebotomus papatasi*; 32 for *Phlebotomus perniciosus*; and 55 for *Phlebotomus sergenti*).

### Climatic data

A series of climate metrics, describing temperature and precipitation in the study area under two climatic scenarios, were obtained from the WorldClim database. These climate metrics are derived from monthly temperature and rainfall values and represent biologically meaningful variables for characterizing species distribution [38].

The WorldClim data layers included 11 temperature and eight precipitation metrics, expressing spatial variations in annual means, seasonality, and extreme or limiting climatic factors with a spatial resolution of about one square kilometer. The data layers were generated through interpolation of average monthly climate data from 4000 weather stations. Climate layers were available for long-term time series (1950–2000), future projections and past conditions.

Climate change projections were simulated by the Canadian Centre for Climate Modelling and Analysis [39] for 2020, 2050 and 2080 under a pessimistic (A2a) and an optimistic (B2a) story-line scenario. A2a describes a highly heterogeneous future world with regionally oriented economies. The main driving forces are a high rate of population growth, increased energy use, land-use changes and slow technological change. The B2a scenario is also regionally oriented but with a general evolution towards environmental protection and social equity. Compared to A2a, B2a has a lower rate of population growth and a smaller increase in gross domestic product, but more diverse technological changes and slower land-use changes [1].

The original 19 predictor variables were reduced to only 5 climate variables by analysis of collinearity [40]. A correlation matrix between the 19 climate variables was performed using ArcGis 10.1 and a pair of variables with a correlation coefficient greater than 0.9 were considered as highly correlated. For highly correlated variables, when possible, we preferred extreme variables (min, max) to mean variables since the biological behavior of the vector is highly affected by extreme climatic conditions. The final dataset included: maximum temperature of warmest month, minimum temperature of coldest month, precipitation of wettest quarter, precipitation of driest quarter and elevation.

### Species distribution model

We used six individual models contributing to the consensus model including two regression techniques (generalized linear models, GLM; generalized additive models, GAM), two machine learning techniques (artificial neural networks, ANNs; maximum entropy, MaxEnt) and two classification techniques (surface range envelop, SRE; random forest, RF).

Generalized linear models [41] are extensions of traditional linear regression models that allow the mean to depend on the explanatory variables through a link function, and the response variable to be any member of a set of distributions called the exponential family, which includes normal, Poisson and binomial distributions, among others.

Generalized additive models [42] blend properties of generalized linear models with additive models. The purpose of generalized additive models is to maximize the quality of prediction of a dependent variable from various distributions, by estimating unspecific (non-parametric) functions of the predictor variables, which are “connected” to the dependent variable *via* a link function.

Artificial neural networks [43] are a machine learning approach. It consists of creating new linear combinations of the input layers called hidden layers; later the created hidden layers are related to a response layer *via* non-linear function [40].

MaxEnt [44] is also a machine learning method that consists in finding the probability distribution of maximum entropy subject to constraints imposed by information derived from the values of the predictor variables in the occurrence points.

The surface range envelop [45] consists of identifying maximum and minimum values for each input variable from the set of occurrence points. Any pixel with all variables falling between these maximum and minimum limits is included within the range.

Random forest [46] is an ensemble classifier algorithm using many decision tree models, which can be used for classification or regression. Different subsets of the training data are selected, with replacement, to train each tree. The remaining training data are used to estimate variables’ error and importance. Class assignment is made by the number of votes from all of the trees and for regression the average of the results is used.

We combined predictions into an “ensemble” by averaging the models with a true skill statistic [47] higher than 0.8 to obtain a “consensus model” and to avoid the integration of weak models. The main aim of consensus methods is to decrease the predictive uncertainty of single-models, since predictions of species distributions can vary widely among modeling approaches. Indeed, ensemble forecasting can enable a more robust model and overcome the uncertainties derived from each individual model [23].

True skill statistic (TSS) includes omission and commission errors. It ranges from -1 to +1, where +1 indicates perfect classification and values below zero indicate a performance no better than random. We used TSS to evaluate the potential species ranges because it has been shown to be insensitive to prevalence and is unaffected by the size of the validation set [47].

As most of the used algorithms need presence/absence data for the model fitting and evaluation, we randomly selected pseudo-absence points in locations where the studied species was not present but the other remaining sand fly species had been recorded, as suggested by Phillips et al. [48] and Mateo et al. [49].

We ran for each model a 5-fold cross-validation by randomly splitting our occurrence data into 70% used for the model calibration and 30% used to evaluate the current predictive performance of the models. This 5-fold cross-validation yielded an average model for each species and algorithm. Calibrated models, for current conditions, were then used to generate projections of future potential distributions for 2020, 2050 and 2080 under the two climate change scenarios, A2a and B2a. For species with no model presenting a TSS score higher than 0.8, no consensus projection was created and the species were removed from further analyses. All analyses were carried out using the *Biomod2* package [50] for R [51].

## Results

**Fig. 1:**
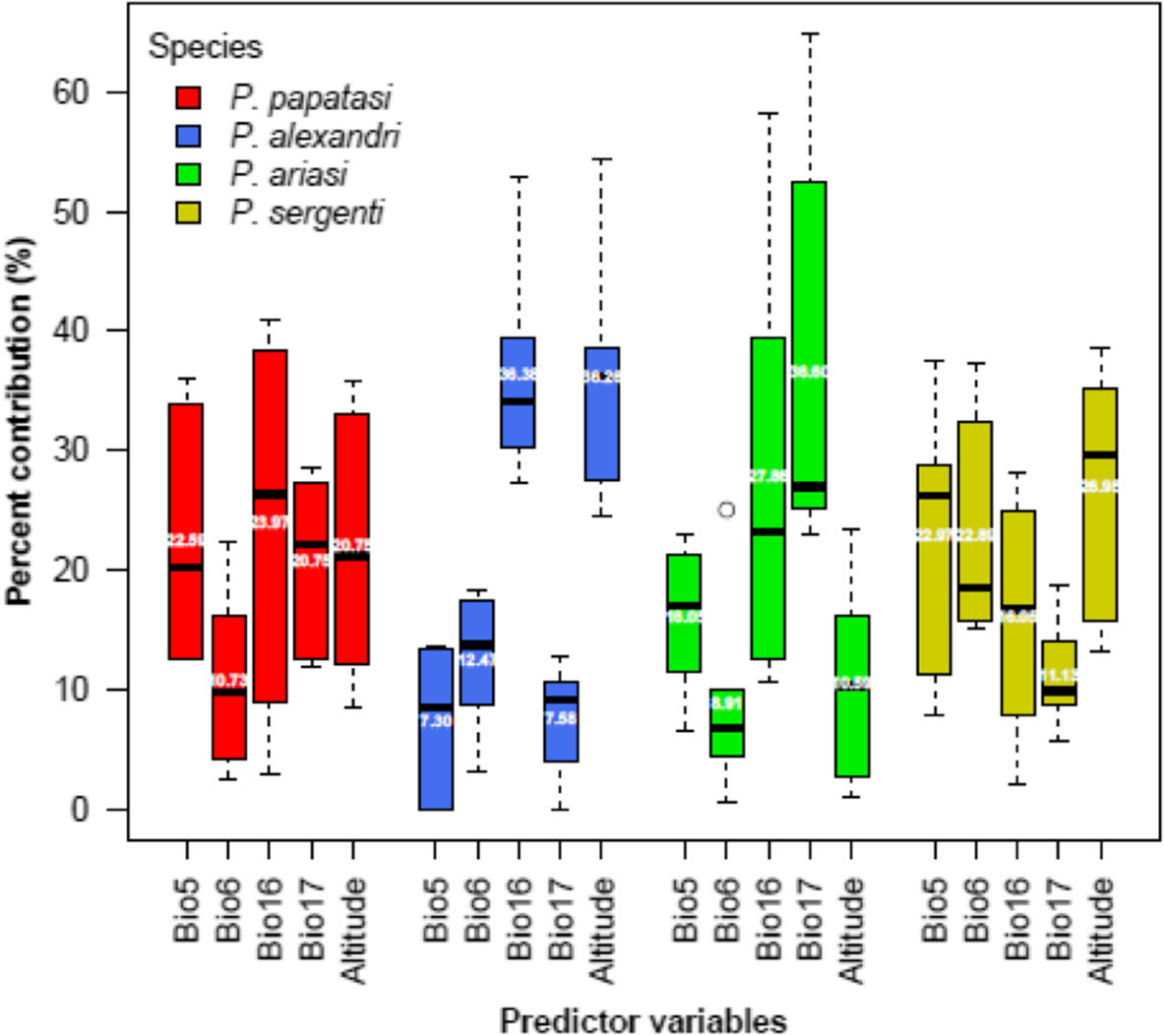
Percent contribution of environmental variables for the final model fitting. *Abbreviations*: Bio5, maximum temperature of warmest month; Bio6, minimum temperature of coldest month; Bio16, precipitation of wettest quarter; Bio17, precipitation of driest quarter

Referring to the mean TSS score (0.84 ± 0.023), model performance was generally high for the included species with a specificity (true negative rate) ranging from 81.03 to 96.52% (mean = 86.94%) and a sensitivity (true positive rate) ranging from 87.93 to 100% (mean = 96.98%). Precipitation of the wettest quarter (Bio16) and precipitation of the driest quarter (Bio17) were the most contributing variables to the models for *P. papatasi*, *P. ariasi* and *P. alexandri* with 44.72, 43.96 and 64.46%, respectively, of explained variation (see Fig. 1). On the other hand, the minimum temperature of the coldest month (Bio6) and the maximum temperature of the warmest month (Bio5) had the highest contribution to the *P. sergenti* model with 45.86% of explained variation. The contribution of the altitude ranged from 10.59% for the *P. ariasi* model to 36.26% for the *P. alexandri* model, with a mean contribution among all modeled species of 23.64%.

**Fig. 2:**
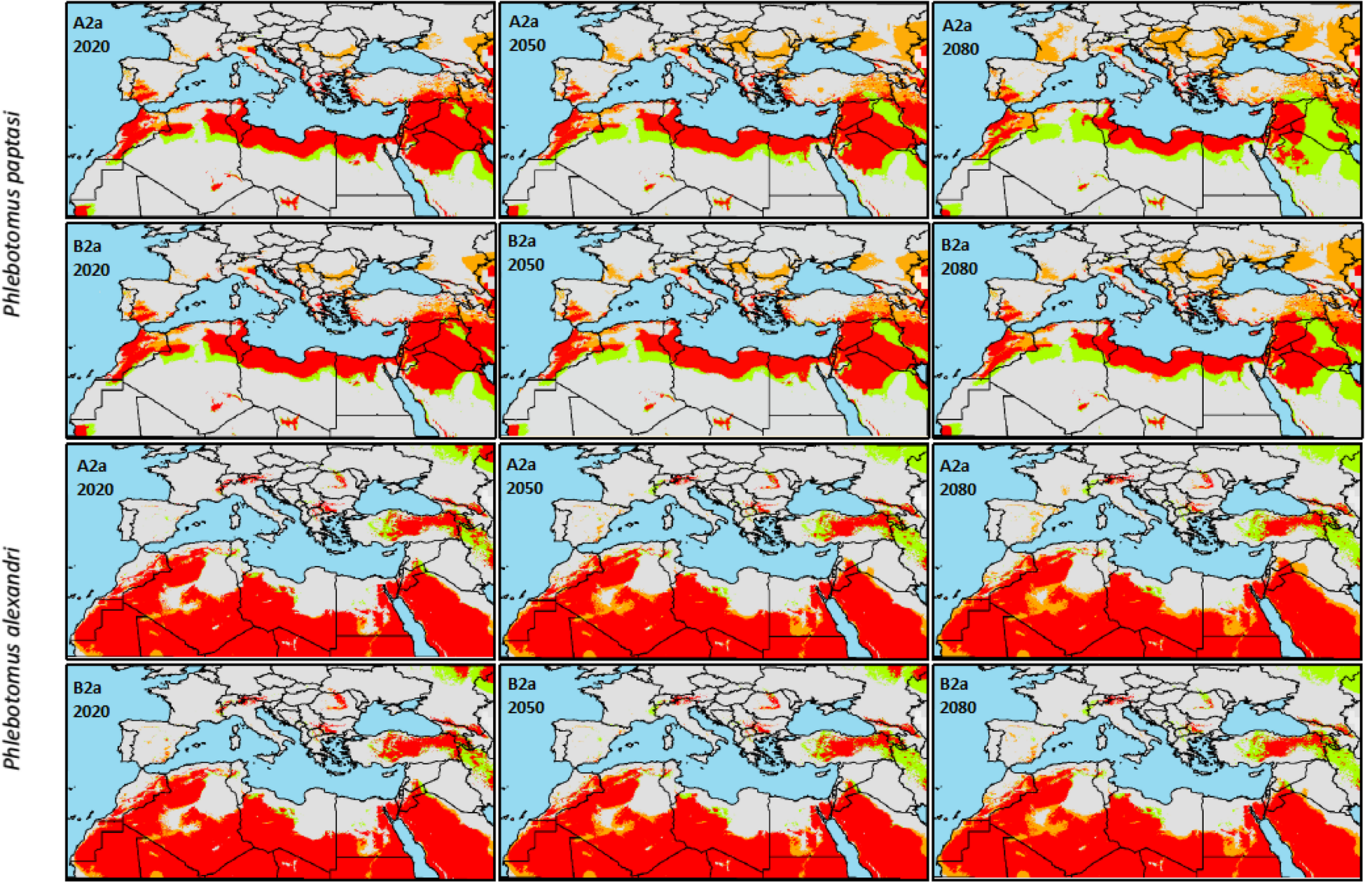
Geographical distribution of *Plebotomus papatasi* and *Plebotomus alexandri* under the pessimistic climate change scenario A2a and the optimistic climate change scenario B2a for the three time periods (2020, 2050 and 2080). Gray color indicates areas predicted to be stable for the species absence, orange color indicates areas to be gained by the species, green color indicates areas to be lost by the species and red color indicates areas to be stable for the species presence

**Fig. 3:**
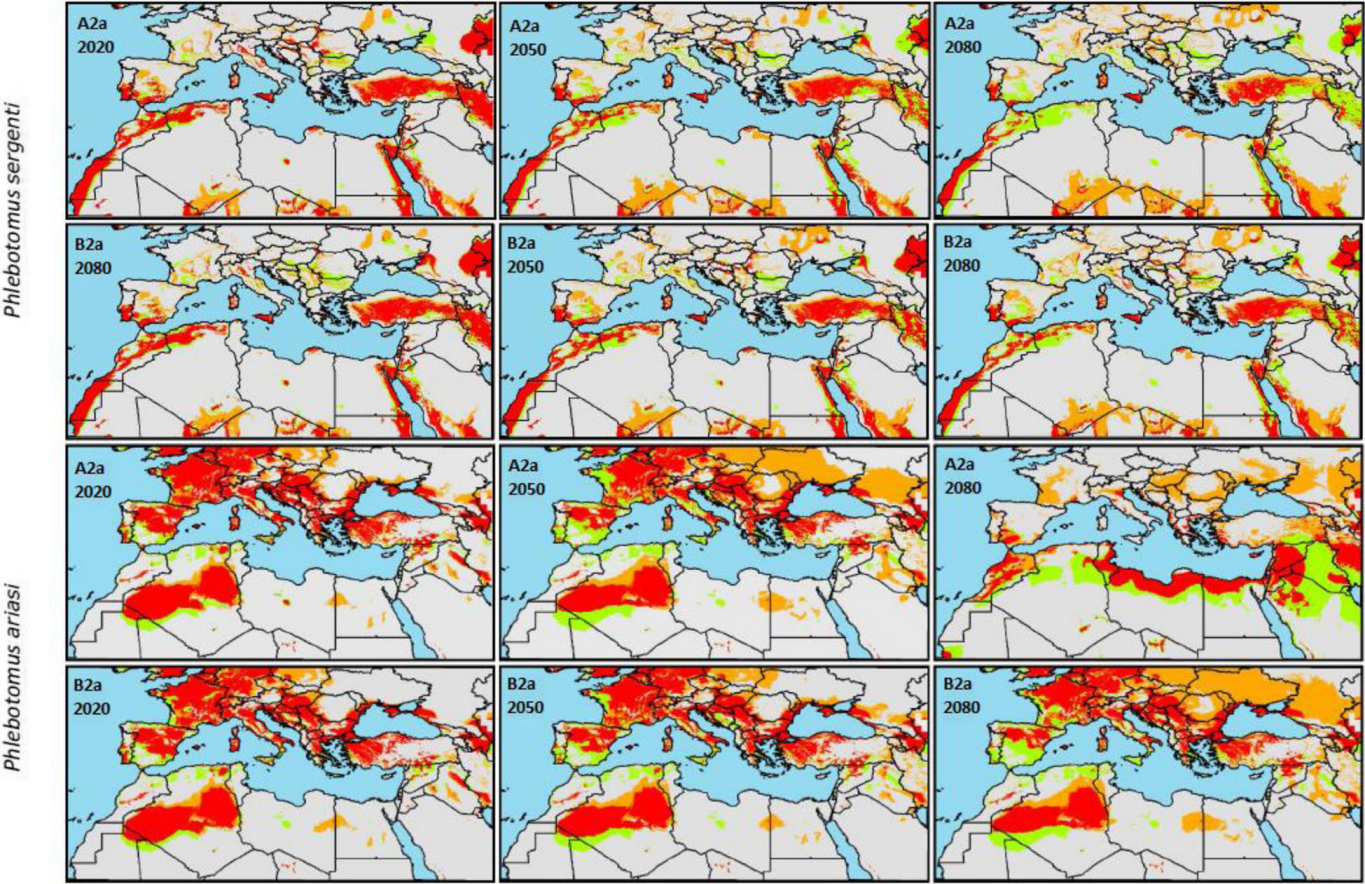
Geographical distribution of *Plebotomus ariasi* and *Plebotomus sergenti* under the pessimistic climate change scenario A2a and the optimistic climate change scenario B2a for the three time periods (2020, 2050 and 2080). Gray color indicates areas predicted to be stable for the species absence, orange color indicates areas to be gained by the species, green color indicates areas to be lost by the species and red color indicates areas to be stable for the species presence

Spatial patterns in the predicted suitable area for sand fly occurrence varied among species, between time periods and climate change scenarios (Figs. 2, 3).

### *Phlebotomus papatasi*

Suitable areas for *P. papatasi* presence are predicted occur in North Africa and the Middle East. Lower occurrence was predicted in the south of most European countries bordering the Mediterranean Sea. The areas suitable for *P. papatasi* range from a latitude below 19°N in the African Sahara to a latitude above 48°N in southern Europe.

Climate change projection for *P. papatasi* showed that the species will shift northward, with a centroid shift estimated at 292, 595 and 948 km for the time periods 2020, 2050 and 2080, respectively, under the A2a (pessimistic) scenario (Table 1). Similar trends were observed for the B2a scenario. However, the shift of the centroid of the suitable area for *P. papatasi* occurrence was lower, with a maximum shift of 728 km northward suspected to occur in 2080.

**Table 1:** Centroid shift, shift direction, conserved area, lost area and gained area in the Mediterranean basin for the different studied phlebotomine species for the three reference years (2020, 2050 and 2080) under the pessimistic scenario A2a and the optimistic scenario B2a

	Centroid shift (km)^a^			Shift direction (°)			Conserved area (%)			Lost area (%)^b^			Gained area (%)		
	2020	2050	2080	2020	2050	2080	2020	2050	2080	2020	2050	2080	2020	2050	2080
Scenario A2a															
*P. papatasi*	292	595	948	17	14	359	84.1	72.5	49.3	-16.0	-27.5	-50.7	21.8	45.2	63.6
*P. alexandri*	341	483	484	232	231	231	90.5	87.0	86.1	-9.5	-13.0	-13.9	4.9	9.1	12.8
*P. ariasi*	408	1019	1279	67	71	75	83.4	71.2	62.5	-16.7	-28.8	-37.6	29.7	69.4	90.5
*P. sergenti*	88	290	255	211	235	211	75.3	56.8	41.3	-14.7	-43.2	-58.7	35.2	51.8	68.1
Scenario B2a															
*P. papatasi*	298	460	728	358	5	13	83.8	77.1	67.9	-16.3	-22.9	-32.1	24.5	32.9	47.1
*P. alexandri*	304	401	533	233	233	233	91.4	88.8	85.8	-8.6	-11.2	-14.2	5.8	7.4	10.1
*P. ariasi*	298	524	1150	69	70	71	82.0	76.6	71.3	-18.1	23.4	-28.7	26.8	37.5	80.3
*P. sergenti*	67	15	155	184	152	233	74.4	67.5	58.6	-25.6	-32.5	-41.4	25.6	50.3	57.8

^a^The centroid shift is the geodesic distance between the centroids of the present and the potential future specie ranges, the shift direction is the angle between North (0°) and the geodesic path between the present and the potential future centroids

^b^The lost area is the percentage of area lost by the species in comparison to the current species range and the gained area is the percentage of area gained by the species in comparison to the current species range

Under the two climate change scenarios (A2a and B2a) and the three time periods (2020, 2050 and 2080) the suitable area for *P. papatasi* presence was predicted to be gained progressively in northern and western Europe. Concurrently, the potential area for species presence would be lost in the south of north African countries and the Middle East (Fig. 2). Nevertheless, the total gained area is higher than the total lost area for all scenarios and time periods (Table 1).

### *Phlebotomus alexandri*

Ecological niche modeling for current conditions showed that the suitable area for *P. alexandri* occurrence is widely distributed from Morocco in the west, to Iran and Kazakhstan in the east of the study area. *Phlebotomus alexandri* is predicted to be less present in western and central Europe, where it was sporadically predicted in Spain, plains bordering the Alps chain and in some countries of central Europe (Fig. 2).

The model projection into the two future climate change scenarios showed that the suitable area for *P. alexandri* distribution will be lost in the north-east of the study area which will cause a centroid shift estimated at 314 and 304 km toward the south-west under A2a and B2a, respectively, in 2020. A higher shift in the same direction is predicted for 2050 and 2080 under both scenarios. For all time-periods and under the two climate change scenarios, the percent of lost area predicted in central and western Asia (especially in Russia, Kazakhstan, Turkey and Iran) was higher than the percent of gained area (Table 1), which was predicted to occur essentially in north African countries, the Middle East, southern Spain and France.

### *Phlebotomus ariasi*

The area with suitable environmental conditions for *P. ariasi* occurrence under current conditions was suspected to occur in North Africa, western Europe and most southern European countries, bordering the Mediterranean Sea.

Under both climate change scenarios and the three time-periods, the species is suspected to progressively lose areas of suitable conditions in western Europe, especially in Spain and France, while new area will be gained by the species eastward including central Europe and western Asia. The area gained by the species is far higher than that predicted to be lost (Fig. 3). Although the two climate change scenarios showed a similar trend, a greater change in the distribution of the lost and gained area by the species is predicted under the A2a scenario, especially during the time-period 2020–2050 (gained area 69.39% under A2a, 37.46% under B2a) and 2050–2080 (lost area -37.60% under A2a, -28.70% under B2a). The change in the suitable area for *P. ariasi* distribution is reflected in the shift of its centroid projected to move eastward with a maximum shift in 2080 estimated to reach 1279 and 1150 km under the A2a and B2a climate change scenarios, respectively (Table 1).

### *Phlebotomus sergenti*

Ecological niche modeling under current climatic conditions showed that suitable area for *P. sergenti* is widely distributed. It ranges from a latitude of 18°N on the south border of the north African countries to a latitude of 51°N in the south of the UK. Nevertheless, suitable areas are not uniformly distributed; the species occurs mainly between 24–48°N in northern Africa, southern Europe, central Europe, the Middle East and western Asia.

All future climate change projections showed that gained area will occur in northern Europe and in countries on the south border of north African countries. These regions actually present a reduced suitable area for *P. sergenti* occurrence which will expand progressively during future time-periods, while large areas currently predicted as suitable for the vector presence will be lost, especially in north African countries, southern Europe, the Middle East and western Asia (Fig. 3).

Despite the large change in the suitable area for *P. sergenti* prospected to occur, confirmed by the gained and lost area by the species under the two climate change scenarios, the centroid shift will reach only a maximum shift of 290 km in 2050 under A2a (Table 1).

## Discussion

Ecological niche modeling has allowed the prediction of the potential distribution of *Leishmania* vectors in the Mediterranean basin. Our study provides evidence for the hypothesis of the widespread *Leishmania* vectors under climate change scenarios. All the studied species are prospected to gain new areas that are actually not suitable for the vectors’ survival.

Indeed, our study corroborates other authors’ findings [12, 52, 53] suggesting that the increase of temperature and humidity, due to the global warming prospected to occur during the 21st century, will affect the distribution of *Leishmania* vectors. As suggested by previous studies [12] phlebotomine sand flies are prospected to invade extra-Mediterranean regions especially western and central Europe. Concurrently, *Leishmania* vectors will lose areas for suitable conditions in North Africa and Middle East. These changes in their geographical distribution are more intense under the A2a scenario than the B2a. Similar projections for the distribution of *P. papatasi*, *P. ariasi* and *P. sergenti* were reported by Trajer et al. [54]. Nevertheless, these authors focused their study only on the distribution of phlebotomine species in Europe, since no presence records in North Africa and Middle East were included.

In a non-endemic location, where the species is suspected to occur, special attention should be taken. Field surveys to detect the presence of *Leishmania* vectors and monitoring cases of disease in these regions, is crucial to prevent the emergence of new disease foci. Additionally, domestic pets (especially dogs) travelling between endemic areas and non-endemic regions suspected to be suitable for the vector disease should be controlled to avoid the risk of transmission. Indeed, in non-endemic regions where the vector is suspected to be present, the introduction of infected dogs will contribute to the establishment of the disease cycle.

Our study focused only on the fundamental ecological niche, which represents the abiotic conditions for the species survival. However, the species is in fact present in a restricted part of the fundamental niche due to biotic interactions. Actually, Hutchinson [55] defined the realized niche as the portion of the fundamental niche from which a species is not excluded due to biotic competition. Furthermore, the dispersal ability of the species is limited by the maximum flight distance of phlebotomine sand flies, estimated as 1000 m [56], and geographical barriers. Indeed, even if the wind plays a major role in the species dissemination, the presence of mountain chains and water bodies can limit their geographical dispersal to new suitable areas for the species’ survival and reproduction [57].

## Conclusions

Climate change is projected to play an essential role in the expansion of the geographical distribution of specific species of leishmaniasis vectors. Sand fly species are prospected to invade extra-Mediterranean regions, especially in Europe. The monitoring of regions presenting suitable conditions for the vectors’ presence is highly recommended to prevent the outbreak of the disease in densely populated areas with a naïve population. Our study confirmed the importance of environmental and climate factors on the distribution of leishmaniasis vectors and demonstrated the performance of ecological niche modeling in the prediction of the geographical spread of vector diseases. Ecological niche modeling should be considered in the future as a valuable tool in addition to experimental laboratory studies for a better understanding of the biology of vector species.

## Abbreviations

ANNs: Artificial neural networks
Bio16: Precipitation of wettest quarter
Bio17: Precipitation of driest quarter
Bio5: Maximum temperature of warmest month
Bio6: Minimum temperature of coldest month
GAM: Generalized additive models
GLM: Generalized linear models
RF: Random Forest
SRE: Surface range envelop
TSS: True skill statistic
